# Technology Dependence Among Hospitalized US Children

**DOI:** 10.1001/jamanetworkopen.2025.9474

**Published:** 2025-05-09

**Authors:** Emily J. Goodwin, Isabella Zaniletti, S. Margaret Wright, Matt Hall, Jeffrey D. Colvin

**Affiliations:** 1 Children’s Mercy Kansas City, Kansas City, Missouri; 2 University of Missouri-Kansas City School of Medicine; 3 University of Kansas Medical Center, Kansas City; 4 Children’s Hospital Association, Lenexa, Kansas

## Abstract

This cross-sectional study assesses the frequency of pediatric inpatient discharges with technology dependence and the most frequent types and combinations of technology dependence across all hospital types.

## Introduction

Children with technology dependence (TD) require a medical device (eg, tracheostomy tube), and if it fails, they would likely require hospitalization.^1^ Caring for children with TD requires caregivers and clinical team expertise. Awareness of the most common TD types and combinations may allow hospitals to prepare and optimize care. However, it is unknown what percentage of pediatric hospitalizations include TD and which types and combinations are most prevalent. Prior researchers have explored the association of TD with complex chronic conditions (CCCs) but have not examined TD at all hospital types, across all TD types, and all CCCs, nor considered combinations of TD.^1,2,3^ The recently published CCC classification system version 3 (CCCv3) includes updated billing codes and TD separated from CCCs, giving a new opportunity to examine TD.^4^ While the CCCv3 aligns TD to 10 body systems (eg, gastrointestinal), it does not categorize TD into clinically distinct types (eg, gastrostomy or cecostomy). Our objective was to determine: (1) the frequency of pediatric inpatient discharges with TD and (2) the most frequent types and combinations of TD across all hospital types using the CCCv3.

## Methods

We performed a retrospective cross-sectional study of the Kids Inpatient Database (KID). KID is the largest pediatric all-payer inpatient database with hospitalizations from 3998 acute care hospitals of all types (not just children’s hospitals) in 49 states.^5^ We excluded admissions for uncomplicated newborns and obstetrics and gynecology conditions. Using CCCv3, we identified hospitalizations with *International Statistical Classification of Diseases, Tenth Revision, Clinical Modification* (*ICD-10-CM*) codes consistent with TD.^4^ Two study team members (E.G. and J.C.) assigned each of the 1279 *ICD-10-CM* codes for TD into 7 body systems and 54 TD categories (eTables 1-3 in Supplement 1). We resolved categorization discrepancies by study team consensus. The statistical analytic system file categorizing the 1279 *ICD-10-CM* codes is available in the eMethods in Supplement 2. We determined the 10 most common prevalent combinations of TD by discharge by absolute rank. We calculated weighted frequencies and compared discharges with and without TD using χ^2^ tests. We summarized results following the STROBE reporting guideline.^6^ The Children’s Mercy Kansas City institutional review board deemed this study non–human participants research, therefore not requiring informed consent. Analysis was from October 2023 to March 2025 using SAS Enterprise Guide version 8.3 (SAS Institute). Statistical significance was a 2-sided *P* < .05.

## Results

Of the 3.5 million weighted discharges, 6.7% (95% CI, 6.1%-7.2%) had 1 or more TDs (Table 1). A higher percentage of discharges with TD had 1 or more non-TD CCCs (83.5%; 95% CI, 83.5%-85% vs 18.8%; 95% CI, 18.7%-20.4%) and relied on Medicaid (55.2%; 95% CI, 52.8%-57.6% vs 49.3%; 95% CI, 48.1%-50.3%) than those with no TD. Among the 234 184 discharges with TD, gastrostomy tubes were most common (113 703 discharges [48.6%]) followed by ventricular shunts (32 582 discharges [13.9%]), tracheostomies (29 223 discharges [12.5%]), and supplemental oxygen (18 199 discharges [7.8%]) (Table 2). Moreover, 44 019 (18.8%) had 2 TDs, with the most common combinations being gastrostomy tubes with either a tracheostomy (8097 discharges [3.5%]), supplemental oxygen (5729 discharges [2.4%]), or a ventricular shunt (5575 discharges [2.4%]). The median (IQR) number of discharges with TD per hospital were significantly higher at urban teaching hospitals compared with rural and urban nonteaching hospitals (13 [4-59] vs 3 [2-4] vs 3 [2-7] discharges; *P* < .001).

**Table 1.** Patient Characteristics of Hospitalizations With and Without Technology Dependence

Characteristic	Participants, No. (%)^a^		
	All hospitalizations (N = 3 512 626)	Technology dependence (n = 234 184)	No technology dependence (n = 3 278 442)
Age, y			
0-4	2 327 244 (66.2)	102 489 (43.8)	2 224 755 (67.9)
5-9	244 823 (7.0)	36 159 (15.4)	208 664 (6.4)
10-14	322 373 (9.2)	40 163 (17.2)	282 210 (8.6)
15-20	618 186 (17.6)	55 373 (23.6)	562 813 (17.1)
Sex			
Female	1 657 323 (47.2)	108 685 (46.4)	1 548 638 (47.3)
Male	1 853 883 (52.8)	125 468 (53.6)	1 728 415 (52.7)
Unknown	1420 (<0.1)	31 (<0.1)	1389 (<0.1)
Race and ethnicity^b^			
Hispanic	665 892 (20.4)	49 077 (22.2)	616 815 (20.3)
Non-Hispanic Black	582 011 (17.9)	38 752 (17.5)	543 259 (17.9)
Non-Hispanic White	1 617 871 (49.7)	111 325 (50.3)	1 506 546 (49.6)
Non-Hispanic other	390 960 (12.0)	22 045 (10.0)	368 915 (12.2)
Unknown	255 892 (7.3)	12 985 (5.5)	242 907 (7.4)
Insurance			
Medicaid	1 741 864 (49.7)	129 185 (55.2)	1 612 679 (49.3)
Private	1 476 949 (42.1)	85 722 (36.6)	1 391 227 (42.5)
Self-pay	151 790 (4.3)	4099 (1.8)	147 691 (4.5)
Medicare	12 302 (0.4)	2760 (1.2)	9542 (0.3)
No charge	4741 (0.1)	198 (0.1)	4543 (0.1)
Other	119 813 (3.4)	11 875 (5.1)	107 938 (3.3)
Unknown	5166 (0.2)	345 (0.15)	4822 (0.2)
No. of complex chronic conditions			
0	2 673 661 (76.1)	36 949 (15.8)	2 636 712 (80.4)
1	534 054 (15.2)	33 795 (14.4)	500 259 (15.3)
≥2	304 911 (8.7)	163 440 (69.8)	141 471 (4.3)
Patient location			
Counties >1 million population	2 013 170 (57.5)	130 162 (56)	1 883 008 (57.6)
Counties 250 000-999 999 population	731 169 (20.9)	50 369 (21.7)	680 800 (20.8)
Counties 50 000-249 999 population	293 679 (8.4)	20 516 (8.8)	273 163 (8.4)
Counties <50 000 population	461 478 (13.2)	31 516 (13.6)	429 962 (13.2)
Unknown	13 130 (0.4)	1621 (0.7)	11 509 (0.4)
Hospital bed size			
Small	520 438 (14.8)	28 705 (12.3)	491 733 (15)
Medium	822 323 (23.4)	45 336 (19.4)	776 987 (23.7)
Large	2 169 865 (61.8)	160 143 (68.4)	2 009 722 (61.3)
Hospital location and teaching status			
Rural	196 758 (5.6)	2541 (1.1)	194 217 (5.9)
Urban nonteaching	373 038 (10.6)	5033 (2.1)	368 005 (11.2)
Urban teaching	2 942 830 (83.8)	226 610 (96.8)	2 716 220 (82.9)

^a^ All group comparisons yielded *P* ≤ .001, with the exception of hospital bed size (*P* = .04).

^b^ Race and ethnicity were collected by participating hospitals, and the method of identifying participant race and ethnicity may vary. The Agency for Healthcare Research and Quality Healthcare Cost and Utilization Project classifies race and ethnicity as Asian or Pacific Islander, Black, Hispanic, Native American, White, and other. In our categorization of race and ethnicity, non-Hispanic other includes Asian or Pacific Islander, Native American, and any race or ethnicity not otherwise specified.

**Table 2.** The Most Frequent TD Types and Combinations Among Pediatric Hospitalizations

Type of TD	All hospitalizations with TD, No. (%) (n = 234 184)	Participants by age, No. (%)			
		0-4 y (n = 102 489)	5-9 y (n = 36 159)	10-14 y (n = 40 163)	15-20 y (n = 55 373)
Gastrostomy tube	113 703 (48.6)	59 209 (57.8)	20 927 (57.9)	16 213 (40.4)	17 354 (31.3)
Ventricular shunt	32 582 (13.9)	13 650 (13.3)	6341 (17.5)	6153 (15.3)	6438 (11.6)
Tracheostomy	29 223 (12.5)	14 467 (14.1)	4807 (13.3)	4070 (10.1)	5879 (10.6)
Supplemental oxygen	18 199 (7.8)	11 920 (11.6)	2275 (6.3)	1942 (4.8)	2062 (3.7)
Other cardiac technology	16 451 (7)	6842 (6.7)	2862 (7.9)	2900 (7.2)	3847 (6.9)
Ostomy (ileostomy or colostomy)	16 092 (6.9)	7713 (7.5)	1913 (5.3)	2025 (5.0)	4441 (8.0)
Spinal fusion	15 859 (6.8)	252 (0.2)	858 (2.4)	6965 (17.3)	7784 (14.1)
Ventilator	15 292 (6.5)	8361 (8.2)	2317 (6.4)	2133 (5.3)	2481 (4.5)
Vascular access device	11 806 (5.0)	5234 (5.1)	1825 (5.0)	1948 (4.8)	2799 (5.1)
Dialysis	8896 (3.8)	2429 (2.4)	1075 (3.0)	1464 (3.6)	3928 (7.1)
Combination of 2 TD types					
Gastrostomy tube and tracheostomy	8097 (3.5)	3935 (3.8)	1445 (4.0)	1009 (2.5)	1708 (3.1)
Gastrostomy tube and supplemental oxygen	5729 (2.4)	3618 (3.5)	885 (2.4)	649 (1.6)	577 (1.0)
Gastrostomy tube and ventricular shunt	5575 (2.4)	2630 (2.6)	1261 (3.5)	926 (2.3)	758 (1.4)
Gastrostomy tube and ostomy (ileostomy or colostomy)	1964 (0.8)	1004 (1.0)	354 (1.0)	274 (0.7)	332 (0.6)
Gastrostomy tube and other cardiac technology	1914 (0.8)	974 (1.0)	404 (1.1)	235 (0.6)	301 (0.5)

Abbreviation: TD, technology dependence.

## Discussion

This cross-sectional study found that approximately 1 in 20 pediatric inpatient discharges had TD, most commonly gastrostomy tubes. Nearly 20% of hospitalizations with TD had 2 types of TD, most commonly gastrostomy and tracheostomy tubes.

Understanding the most common TD types and combinations during admissions is important for hospital preparedness for children with TD, including training on TD use and complications, stocking related equipment, and colocating nursing with TD expertise. We found that pediatric hospitalizations with TD were uncommon at rural hospitals, which may have implications for the preparedness of those hospitals to treat rural-residing children with TD.^3^

Our findings are limited to inpatient hospitalizations and exclude observation status. Due to the nature of our data source, we cannot distinguish between TD utilized solely during hospitalization. Because children with CCCs have high hospitalization rates, the proportion with TD in the outpatient setting is likely lower. The percentage of TD hospitalizations with dependence on supplemental oxygen was lower than expected based on our clinical experience. The coding of dependence on supplemental oxygen deserves further investigation and validation. Future researchers can use the categorization of TD codes to examine the prevalence of TD.

## Notes

Supplement 1. **eTable 1.** TD by Body System **eTable 2.** TD Category Description **eTable 3.** TD Categories

Supplement 2. **eMethods.** Statistical Analytic System File

Supplement 3.
Data Sharing Statement
