# Supplementary material for: Technology Dependence Among Hospitalized US Children
Source: JAMA Netw Open. 2025 May 9;8(5):e259474. doi: 10.1001/jamanetworkopen.2025.9474 (PMC12065039; doi:10.1001/jamanetworkopen.2025.9474)
Supplement: Supplement 2. — eMethods. Statistical Analytic System File [file jamanetwopen-e259474-s002.pdf]

Supplemental Online Content

Goodwin EJ, Zaniletti I, Wright SM, Hall M, Colvin JD. Technology dependence among hospitalized US children. *JAMA Network Open*. 2025;8(5):e259474.
doi:10.1001/jamanetworkopen.2025.9474

eMethods. Statistical Analytic System File

This supplemental material has been provided by the authors to give readers additional information about their work.

© 2025 Goodwin EJ et al. *JAMA Network Open*.

eMethods. Statistical Analytic System File

```
/***************************************************************************
**********************
Macro Name: Tech_Dep_v1

Function:
This program will be used to create Tech dependent categories flag and
compute the number of TD category

Author: Isabella Zaniletti, PhD MA; Children's Hospital Association

Date: November 26, 2024

Load macro:

%INCLUDE "\path\Tech_Dep_v1.sas";

Call statement:

%Tech_Dep_v1(dt_in,dt_out,dx,n_dxs,pr,n_prs);

Parameter definitions:

   dt_in:  SAS input data set containing patient id and all ICD-10-CM codes
   dt_out: SAS output data set containing all input data and created
individual TD flags,
           overall at least one TD flag (TD_flag), number of TD (num_TD), and
body system categories
   dx: prefix for ICD-10-CM diagnosis code
   n_dxs: number  ICD-10-CM diagnosis code
   pr: prefix for ICD-10-CM procedure code
   n_prs: number  ICD-10-CM procedure code


***************************************************************************
*********************/

*PATHNAME specifies the location of the Excel file "Tech_Dep_codes_v1.xlsx";

%LET PATHNAME=\\pr33e\SAS\Jobs\isabella.zaniletti\PHIS\JEFFREY\A PAPER IN A
DAY\EMILY TECH DEP\For manuscript;  *<===USER MUST modify;

/*Create TD Body System Category Formats*/
PROC IMPORT FILE="&PATHNAME.\Supplement eTable Technology Dependence
Categories.xlsx" DBMS=xlsx OUT=TD_V1 REPLACE;
        SHEET="TD Categories";
RUN;
DATA TD_V1;
        SET TD_V1;
        TD_category = COMPRESS(TD_categories, , 'kas');
RUN;
PROC SQL;
        CREATE TABLE TD_categories AS
        SELECT DISTINCT ICD_Code, DX_PR, UPCASE(TD_category) AS TD_category
        FROM TD_V1
        ORDER BY TD_category;
QUIT;
```

© 2025 Goodwin EJ et al. *JAMA Network Open*.

```
DATA TD_bs_dxs TD_bs_prs;
        FORMAT fmtname $30.;
        SET TD_categories;
        IF DX_PR='DX' THEN fmtname=COMPRESS('$'||TD_category||'DX');
                ELSE IF DX_PR='PR' THEN
fmtname=COMPRESS('$'||TD_category||'PR');
        IF DX_PR='DX' THEN OUTPUT TD_bs_dxs;
                ELSE OUTPUT TD_bs_prs;
        KEEP ICD_Code TD_category fmtname;
        RENAME ICD_Code=start TD_category=label;
RUN;
/*Combine diagnosis formats & output*/
DATA TD_dx1;
        LENGTH label $50;
        SET TD_bs_dxs ;
        default=12;
RUN;
PROC SORT DATA=TD_dx1;
        BY fmtname;
RUN;
PROC FORMAT LIBRARY=work CNTLIN=TD_dx1;
RUN;
/*Combine procedure formats & output*/
DATA TD_pr1;
        LENGTH label $50;
        SET TD_bs_prs ;
        default=12;
RUN;
PROC SORT DATA=TD_pr1;
        BY fmtname;
RUN;
PROC FORMAT LIBRARY=work CNTLIN=TD_pr1;
RUN;

/*Subcategory Format*/
%MACRO Tech_Dep_v1(dt_in,dt_out,dx,n_dxs,pr,n_prs);
        DATA &dt_out.;
                SET &dt_in.;
                ARRAY dxs(&n_dxs.) $12 &dx.1 - &dx.&n_dxs.;
                ARRAY dxc(&n_dxs.) $50 dxc1-dxc&n_dxs.;
                ARRAY prs(&n_prs.) $12 &pr.1 - &pr.&n_prs.;
                ARRAY prc(&n_prs.) $50 prc1-prc&n_prs.;
                TD01=0; TD02=0; TD03=0; TD04=0; TD05=0; TD06=0; TD07=0; TD08=0;
        TD09=0; TD10=0;
                TD11=0; TD12=0; TD13=0; TD14=0; TD15=0; TD16=0; TD17=0; TD18=0;
        TD19=0; TD20=0;
                TD21=0; TD22=0; TD23=0; TD24=0; TD25=0; TD26=0; TD27=0; TD28=0;
        TD29=0; TD30=0;
                TD31=0; TD32=0; TD33=0; TD34=0; TD35=0; TD36=0; TD37=0; TD38=0;
        TD39=0; TD40=0;
                TD41=0; TD42=0; TD43=0; TD44=0; TD45=0; TD46=0; TD47=0; TD48=0;
        TD49=0; TD50=0;
                TD51=0; TD52=0; TD53=0; TD54=0;
                cvd_TD=0; gi_TD=0; metab_TD=0; misc_TD=0; neuro_TD=0;
renal_TD=0; respiratory_TD=0; TD_flag=0;

                /*ICD 10 Version*/
```

© 2025 Goodwin EJ et al. *JAMA Network Open*.

```
                        DO I = 1 TO &n_dxs.;
                                IF dxs(I)~='' THEN DO;
                                        dxc(I)=PUT(dxs(I),$ARTIFICIALHEARTDX.);
IF dxc(I)~=dxs(I) THEN TD01=1;

        dxc(I)=PUT(dxs(I),$CORONARYBYPASSGRAFTCARDIACDX.); IF dxc(I)~=dxs(I)
THEN TD02=1;

        dxc(I)=PUT(dxs(I),$HEARTASSISTDEVICEDX.); IF dxc(I)~=dxs(I) THEN
TD03=1;
                                        dxc(I)=PUT(dxs(I),$HEARTVALVEDX.); IF
dxc(I)~=dxs(I) THEN TD04=1;
                                        dxc(I)=PUT(dxs(I),$OTHERCARDIACDX.); IF
dxc(I)~=dxs(I) THEN TD07=1;

        dxc(I)=PUT(dxs(I),$PACEMAKERORDEFIBRILLATORDX.); IF dxc(I)~=dxs(I)
THEN TD08=1;


        dxc(I)=PUT(dxs(I),$FEEDINGDEVICEGASTROSTOMYDX.); IF dxc(I)~=dxs(I)
THEN TD18=1;
                                        dxc(I)=PUT(dxs(I),$GASTRICBANDDX.); IF
dxc(I)~=dxs(I) THEN TD19=1;

        dxc(I)=PUT(dxs(I),$OSTOMYILEOSTOMYORCOLOSTOMYDX.); IF dxc(I)~=dxs(I)
THEN TD21=1;

        dxc(I)=PUT(dxs(I),$OTHERGASTROINTESTINALDX.); IF dxc(I)~=dxs(I) THEN
TD23=1;

                                        dxc(I)=PUT(dxs(I),$INSULINPUMPDX.); IF
dxc(I)~=dxs(I) THEN TD28=1;

                                        dxc(I)=PUT(dxs(I),$OTHEREXTREMITYDX.);
IF dxc(I)~=dxs(I) THEN TD29=1;

        dxc(I)=PUT(dxs(I),$OTHERIMPLANTEDDEVICEDX.); IF dxc(I)~=dxs(I) THEN
TD30=1;
                                        dxc(I)=PUT(dxs(I),$OTHERSTOMADX.); IF
dxc(I)~=dxs(I) THEN TD31=1;


        dxc(I)=PUT(dxs(I),$INFUSIONPUMPCRANIALORSPINALDX.); IF dxc(I)~=dxs(I)
THEN TD33=1;

        dxc(I)=PUT(dxs(I),$OTHERNERVOUSSYSTEMDEVICEDX.); IF dxc(I)~=dxs(I)
THEN TD35=1;
                                        dxc(I)=PUT(dxs(I),$STIMULATORNEURODX.);
IF dxc(I)~=dxs(I) THEN TD37=1;

        dxc(I)=PUT(dxs(I),$VENTRICULARSHUNTDX.); IF dxc(I)~=dxs(I) THEN
TD40=1;

                                        dxc(I)=PUT(dxs(I),$DIALYSISDX.); IF
dxc(I)~=dxs(I) THEN TD43=1;
                                        dxc(I)=PUT(dxs(I),$VESICOSTOMYDX.); IF
dxc(I)~=dxs(I) THEN TD48=1;
```

© 2025 Goodwin EJ et al. *JAMA Network Open*.

```
        dxc(I)=PUT(dxs(I),$OTHERRESPIRATORYDX.); IF dxc(I)~=dxs(I) THEN
TD50=1;
                                      dxc(I)=PUT(dxs(I),$OXYGENDX.); IF
dxc(I)~=dxs(I) THEN TD51=1;
                                      dxc(I)=PUT(dxs(I),$TRACHEOSTOMYDX.); IF
dxc(I)~=dxs(I) THEN TD53=1;
                                      dxc(I)=PUT(dxs(I),$VENTILATORDX.); IF
dxc(I)~=dxs(I) THEN TD54=1;
                              END;
                      END;

                      DO J = 1 TO &n_prs.;
                              IF prs(J)~='' THEN DO;
                                      prc(J)=PUT(prs(J),
$HEARTASSISTDEVICEPR.); IF prc(J)=prs(J) THEN prc(J)=""; ELSE TD03=1;
                                      prc(J)=PUT(prs(J),
$INTERAORTICPUMPPR.); IF prc(J)=prs(J) THEN prc(J)=""; ELSE TD05=1;
                                      prc(J)=PUT(prs(J),
$INTERNALMONITORINGDEVPR.); IF prc(J)=prs(J) THEN prc(J)=""; ELSE TD06=1;
                                      prc(J)=PUT(prs(J),
$PACEMAKERORDEFIBRILLATORPR.); IF prc(J)=prs(J) THEN prc(J)=""; ELSE TD08=1;
                                      prc(J)=PUT(prs(J),
$STIMULATORCHESTPR.); IF prc(J)=prs(J) THEN prc(J)=""; ELSE TD09=1;

                                      prc(J)=PUT(prs(J),
$BYPASSESOPHAGEALPR.); IF prc(J)=prs(J) THEN prc(J)=""; ELSE TD10=1;
                                      prc(J)=PUT(prs(J), $BYPASSSTOMACHPR.);
IF prc(J)=prs(J) THEN prc(J)=""; ELSE TD11=1;
                                      prc(J)=PUT(prs(J), $CECOSTOMYPR.); IF
prc(J)=prs(J) THEN prc(J)=""; ELSE TD12=1;
                                      prc(J)=PUT(prs(J), $DILATIONGIPR.); IF
prc(J)=prs(J) THEN prc(J)=""; ELSE TD13=1;
                                      prc(J)=PUT(prs(J),
$DRAINGASTROINTESTINALPR.); IF prc(J)=prs(J) THEN prc(J)=""; ELSE TD14=1;
                                      prc(J)=PUT(prs(J),
$ESOPHAGEALDEVICEPR.); IF prc(J)=prs(J) THEN prc(J)=""; ELSE TD15=1;
                                      prc(J)=PUT(prs(J),
$FEEDINGDEVICEESOPHAGEALPR.); IF prc(J)=prs(J) THEN prc(J)=""; ELSE TD16=1;
                                      prc(J)=PUT(prs(J),
$FEEDINGDEVICEGASTROJEJUNOSTOM.); IF prc(J)=prs(J) THEN prc(J)=""; ELSE
TD17=1;
                                      prc(J)=PUT(prs(J),
$FEEDINGDEVICEGASTROSTOMYPR.); IF prc(J)=prs(J) THEN prc(J)=""; ELSE TD18=1;
                                      prc(J)=PUT(prs(J),
$IRRIGATIONGASTROINTESTINALPR.); IF prc(J)=prs(J) THEN prc(J)=""; ELSE
TD20=1;
                                      prc(J)=PUT(prs(J),
$OSTOMYILEOSTOMYORCOLOSTOMYPR.); IF prc(J)=prs(J) THEN prc(J)=""; ELSE
TD21=1;
                                      prc(J)=PUT(prs(J),
$OTHERABDOMINALSTOMAREPAIRPR.); IF prc(J)=prs(J) THEN prc(J)=""; ELSE TD22=1;
                                      prc(J)=PUT(prs(J),
$OTHERGASTROINTESTINALPR.); IF prc(J)=prs(J) THEN prc(J)=""; ELSE TD23=1;
                                      prc(J)=PUT(prs(J),
$STIMULATORABDOMENPR.); IF prc(J)=prs(J) THEN prc(J)=""; ELSE TD24=1;
```

© 2025 Goodwin EJ et al. *JAMA Network Open*.

```
                                        prc(J)=PUT(prs(J),
$INFUSIONPUMPNONBACKABDOMENPR.); IF prc(J)=prs(J) THEN prc(J)=""; ELSE
TD25=1;
                                        prc(J)=PUT(prs(J),
$INFUSIONPUMPABDOMENPR.); IF prc(J)=prs(J) THEN prc(J)=""; ELSE TD26=1;
                                        prc(J)=PUT(prs(J),
$INFUSIONPUMPBACKPR.); IF prc(J)=prs(J) THEN prc(J)=""; ELSE TD27=1;

                                        prc(J)=PUT(prs(J),
$DRAINAGEOFCEREBRALVENTRICLEPR.); IF prc(J)=prs(J) THEN prc(J)=""; ELSE
TD32=1;
                                        prc(J)=PUT(prs(J),
$IRRIGATIONCRANIALPR.); IF prc(J)=prs(J) THEN prc(J)=""; ELSE TD34=1;
                                        prc(J)=PUT(prs(J), $SPINALFUSIONPR.);
IF prc(J)=prs(J) THEN prc(J)=""; ELSE TD36=1;
                                        prc(J)=PUT(prs(J),
$STIMULATORNEUROPR.); IF prc(J)=prs(J) THEN prc(J)=""; ELSE TD37=1;
                                        prc(J)=PUT(prs(J),
$STIMULATORSTOMACHPR.); IF prc(J)=prs(J) THEN prc(J)=""; ELSE TD38=1;
                                        prc(J)=PUT(prs(J),
$STIMULATORTRUNKPR.); IF prc(J)=prs(J) THEN prc(J)=""; ELSE TD39=1;
                                        prc(J)=PUT(prs(J),
$VENTRICULARSHUNTPR.); IF prc(J)=prs(J) THEN prc(J)=""; ELSE TD40=1;
                                        prc(J)=PUT(prs(J),
$VERTERBROPLASTYPR.); IF prc(J)=prs(J) THEN prc(J)=""; ELSE TD41=1;

                                        prc(J)=PUT(prs(J), $BYPASSRENALPR.); IF
prc(J)=prs(J) THEN prc(J)=""; ELSE TD42=1;
                                        prc(J)=PUT(prs(J), $DIALYSISPR.); IF
prc(J)=prs(J) THEN prc(J)=""; ELSE TD43=1;
                                        prc(J)=PUT(prs(J), $DRAINRENALGUPR.);
IF prc(J)=prs(J) THEN prc(J)=""; ELSE TD44=1;
                                        prc(J)=PUT(prs(J),
$IRRIGATIONRENALGUPR.); IF prc(J)=prs(J) THEN prc(J)=""; ELSE TD45=1;
                                        prc(J)=PUT(prs(J),
$OTHERRENALPROCEDUREPR.); IF prc(J)=prs(J) THEN prc(J)=""; ELSE TD46=1;
                                        prc(J)=PUT(prs(J),
$VASCULARACCESSDEVICEPR.); IF prc(J)=prs(J) THEN prc(J)=""; ELSE TD47=1;
                                        prc(J)=PUT(prs(J), $VESICOSTOMYPR.); IF
prc(J)=prs(J) THEN prc(J)=""; ELSE TD48=1;

                                        prc(J)=PUT(prs(J),
$IRRIGATIONRESPIRATORYPR.); IF prc(J)=prs(J) THEN prc(J)=""; ELSE TD49=1;
                                        prc(J)=PUT(prs(J),
$PACEMAKERDIAPHRAGMPR.); IF prc(J)=prs(J) THEN prc(J)=""; ELSE TD52=1;
                                        prc(J)=PUT(prs(J), $TRACHEOSTOMYPR.);
IF prc(J)=prs(J) THEN prc(J)=""; ELSE TD53=1;
                       END;
               END;

               IF MAX(TD01,TD02,TD03,TD04,TD05,TD06,TD07,TD08,TD09)=1 THEN
cvd_TD=1;
               IF
MAX(TD10,TD11,TD12,TD13,TD14,TD15,TD16,TD17,TD18,TD19,TD20,TD21,TD22,TD23,TD2
4)=1 THEN gi_TD=1;
               IF MAX(TD25,TD26,TD27,TD28)=1 THEN metab_TD=1;
```

© 2025 Goodwin EJ et al. *JAMA Network Open*.

```
                IF MAX(TD29,TD30,TD31)=1 THEN misc_TD=1;
                IF MAX(TD32,TD33,TD34,TD35,TD36,TD37,TD38,TD39,TD40,TD41)=1
THEN neuro_TD=1;
                IF MAX(TD42,TD43,TD44,TD45,TD46,TD47,TD48)=1 THEN renal_TD=1;
                IF MAX(TD49,TD50,TD51,TD52,TD53,TD54)=1 THEN respiratory_TD=1;

                num_TD=SUM(OF TD01-TD54);
                IF num_TD>0 THEN TD_flag=1;

                DROP I J dxc1-dxc&n_dxs. prc1-prc&n_prs.;
        RUN;
%MEND;

/*Clean up*/
PROC DATASETS;
        DELETE TD_v1 TD_bs_dxs TD_bs_prs TD_categories TD_dx1 TD_pr1 ;
RUN; QUIT;
```

© 2025 Goodwin EJ et al. *JAMA Network Open*.
