# Supplementary material for: Technology Dependence Among Hospitalized US Children
Source: JAMA Netw Open. 2025 May 9;8(5):e259474. doi: 10.1001/jamanetworkopen.2025.9474 (PMC12065039; doi:10.1001/jamanetworkopen.2025.9474)
Supplement: Supplement 3. — Data Sharing Statement [file jamanetwopen-e259474-s003.pdf]

Data Sharing Statement

Goodwin. Technology Dependence Among Hospitalized US Children. *JAMA Netw Open*. Published May 09, 2025. doi:10.1001/jamanetworkopen.2025.9474

Data
Data available: No

Additional Information
Explanation for why data not available: The data is publicly available from the Healthcare Cost and Utilization Project (https://hcup-us.ahrq.gov/). The authors are restricted from sharing the data.
